# Combined effect of unsaturated fatty acids and saturated fatty acids on the metabolic syndrome: tehran lipid and glucose study

**DOI:** 10.1186/s41043-015-0015-z

**Published:** 2015-07-11

**Authors:** Somayeh Hosseinpour-Niazi, Parvin Mirmiran, Arefeh Fallah-ghohroudi, Fereidoun Azizi

**Affiliations:** 1 Nutrition and Endocrine Research center, Research Institute for Endocrine Sciences, Shahid Beheshti University of Medical Sciences, Tehran, Iran; 2 Department of Clinical Nutrition and Dietetics, Faculty of Nutrition Sciences and Food Technology, National Nutrition and Food Technology Research Institute, Shahid Beheshti University of Medical Sciences, Tehran, Iran; 3 Endocrine Research Center, Research Institute for Endocrine Sciences, Shahid Beheshti University of Medical Sciences, Tehran, Iran

**Keywords:** Metabolic syndrome, Monounsaturated fatty acid, Polyunsaturated fatty acid, Saturated fatty acid, Interaction

## Abstract

**Aims:**

The aim of this study was to investigate whether the background intakes of total dietary fat, monounsaturated fatty acids (MUFA) and polyunsaturated fatty acids (PUFA) modulate the effects of dietary saturated fatty acids (SFA) on metabolic syndrome (MetS).

**Material and methods:**

This population-based cross-sectional study was conducted on a representative sample of 4 677 adults, aged 19 to 84 years. MetS was defined according to the ATP III criteria.

**Results:**

Median intakes of SFA, MUFA and PUFA were 9.5, 9.6 and 5.6% of total energy. High SFA intakes were associated with higher prevalence of MetS, in both individuals with higher and lower median intakes of total fat, MUFA and PUFA.

**Conclusions:**

Our findings indicate that SFA intakes were positively associated with the prevalence of MetS, independent of total dietary fat, MUFA and PUFA intake.

## Background

Metabolic syndrome (MetS), a constellation of metabolic abnormalities including glucose intolerance, abdominal obesity, dyslipidemia and hypertension [1], is highly prevalent in Iran. With a prevalence of over 30 % in the adult population, this epidemic is foreseen to continue to escalate over the next decade [2]. Health problems associated with this syndrome include diabetes and cardiovascular disease [3]. Although the optimal dietary pattern to reduce progression of MetS has not been well established, a reduction in the proportion of calories from fat, particularly saturated fatty acids (SFA) is generally recommended [4]. Previous studies suggest that reducing the consumption of SFA may be more effective in the prevention of cardiometabolic risk factors [5, 6]. However, the results of recent studies have been conflicting regarding the relative effect of SFA intake on cardiometabolic risk factors; some report no effect [7, 8], while others found a beneficial effect [9, 10]. Background intake of dietary monounsaturated fatty acids (MUFA) and polyunsaturated fatty acids (PUFA) may affect these associations [10, 11]. A recent prospective study investigating the interaction between nutrients and risk of coronary atherosclerosis found that intake of SFA was associated with less progression of coronary atherosclerosis, an association that was significant only among subjects consuming less MUFA [10]. Also in a clinical trial, after categorizing the subjects by the median energy fat percentage, no significant difference was found in high fat diets compared with low fat diets, between the effect of the high SFA and high MUFA diets on insulin sensitivity [12]. An experimental study showed that dietary unsaturated fatty acids interfere with SFA in expression of inflammatory markers [13].

However, previous studies have shown no significant interaction between dietary SFA and PUFA on cardiometabolic risk factors [10, 11]. In a clinical trial study, fish oil had a beneficial effect on some lipid profiles, regardless of whether the diet contained high or low amounts of SFA [11]. Considering the limited data available on the association between interaction of SFA, MUFA, PUFA and MetS, the aim of this study was to investigate whether background intakes of total dietary fat, MUFA and PUFA modulate the effects of dietary SFA on the MetS and its components, among 19–84 year old subjects, participants of the Tehran Lipid and Glucose Study (TLGS).

## Methods

**Fig. 1:**
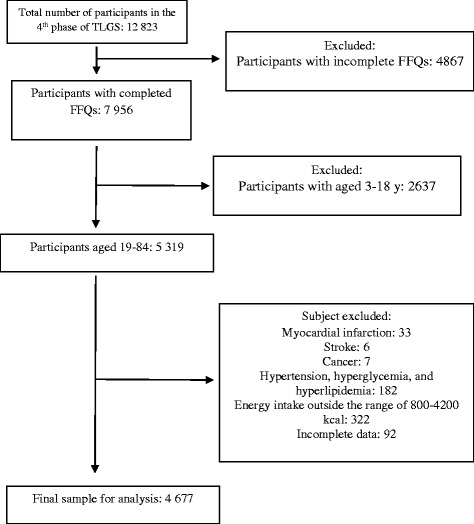
Outline of the selection design of study participants

This population based cross-sectional study, conducted within the framework of the TLGS, an ongoing community-based prospective investigation, included a sample of residents under the coverage of 3 medical health centers in District No. 13 of Tehran, the capital city of Iran. The design of the study has been described previously [14]. Briefly, using multistage cluster random sampling methods, 15 005 people, aged ≥ 3 years, were selected and followed up every 3 years. During the fourth phase of the TLGS (2008–2011), a total of 12 823 subjects completed the examinations and were invited to complete the food frequency questionnaire (FFQ); of the 7 956 who agreed to participate and completed the FFQ, 5 319 were aged 19–84 years. Participants were excluded if they were on any specific diets due to medical history of myocardial infarction (*n* = 33), stroke (n = 6), cancer (n = 7), reported daily energy intakes outside the range of 800–4200 kcal/d (*n* = 322) and for missing data on physical activity, or any of anthropometrical measurements, and biochemical variables (*n* = 92). Also excluded were participants with hyperlipidemia, hyperglycemia and hypertension that had changed their dietary intake (*n* = 182). Finally, data for 4677 individuals (2075 males and 2602 females) were analyzed (Fig. 1). The design of the study was approved by the ethical committee of the Research Institute for Endocrine Sciences, Shahid Beheshti University of Medical Sciences, and informed written consent was obtained from all participants.

### Dietary assessment

Usual dietary intake was assessed using a 147-item validated semi-quantitative FFQ. The validity and reliability of the FFQ have been described in detail elsewhere [15]. The FFQ consists of a list of foods with a standard serving size, commonly consumed by Iranians. Trained dieticians with at least 5 years of experience in the TLGS survey completed the FFQ during face-to-face interviews, asking participants to report their frequency of consumption of a given serving of each food item during the previous year on a daily, weekly, or monthly basis [16]. Details on dietary fat intakes, i.e. type of foods consumed e.g. full fat vs. fat free were obtained. The reported frequency for each food item was converted to a daily intake. Portion sizes of consumed food were then converted to grams using household measures [17]. Energy of each gram of food was obtained from the US Department of Agriculture’s (USDA) Food Composition Table (FCT) by multiplying the gram of consumption of each food by the content of energy per 100 g and total energy intake was calculated by summing up energy intakes from all foods. Composition values for SFA, MUFA and PUFA were obtained from the USDA FCT, because the Iranian FCT with regard to fatty acid intakes is incomplete. Among food items in the FFQ, only ‘*kashk’* was not listed in the USDA FCT, and its content of total fatty acid and SFA was determined from the nutritional facts given for this product.

Validity and reliability of the FFQ were assessed in a random sample of 132 subjects, aged 20 y and over. The validity and reliability of the FFQ for total dietary fat was acceptable [18]; correlation coefficient between the FFQ and multiple 24-h recalls was 0.59 and 0.38; and between the two FFQs was 0.43 and 0.42, in males and females, respectively [15]. Also intraclass correlations between the two FFQs and between multiple 24 recalls and the FFQ for SFA, MUFA and PUFA were acceptable in both genders (range 0.51 to 0.74).

### Biochemical assessment

After 12 to 14 h of overnight fasting, blood samples were drawn into vacutainer tubes in a sitting position, from all study participants. All blood analyses were done at the TLGS research laboratory on the day of blood collection, using a Selectra 2 autoanalyzer (Vita; Scientific, Spankeren, the Netherlands). Serum triglyceride concentrations were measured using triglyceride kits (Pars Azmoon Inc., Tehran, Iran) by the enzymatic calorimetric test with glycerol phosphate oxidase. HDL cholesterol was measured after precipitation of the apolipoprotein B-containing lipoproteins with phosphotungistic acid. Serum fasting glucose concentration was assayed using an enzymatic colorimetric method with the glucose oxidase technique. Inter- and intra-assay coefficients of variations were both 2.2 % for serum glucose, 2 and 0.5 % for HDL-C and 1.6 and 0.6 % for triglycerides, respectively.

### Assessment of other variables

Weight was measured while the subjects were minimally clothed and not wearing shoes, using digital scale and was recorded to the nearest 100 g. Height was measured while subjects were standing without shoes, with their shoulders in a normal position, using a tape fixed to the wall and was recorded to the nearest 0.5 cm. Body mass index (BMI) was calculated as weight (kg) divided by square of height (m^2^). Waist circumference (WC) was measured at the level of the umbilicus site, using an outstretched tape meter, without pressure to body surfaces and was recorded to the nearest 0.5 cm.

Systolic and diastolic blood pressure was measured using a standard mercury sphygmomanometer, on the right arm after a 15-min rest in a sitting position. Two measurements were taken at 1-min intervals and the average of measurement was recorded as the participant’s blood pressure. Physical activity was assessed using a questionnaire, including a list of common activities of daily life; the frequency and amount of time of activities spent per week over the past 12 month were documented [19]. Levels of physical activity were expressed as metabolic equivalent hours per week (METs h/week) [20] and were categorized as light (>3 METs h/week), moderate (3–6 METs h/week) and heavy (≥6 METs h/week) [20]. Cigarette smoking status was categorized as current smoker, non-smoker and ex-smoker. Additional covariate information including age, medical history, and current use of medications was obtained using an interview questionnaire.

### Definition of MetS

MetS was defined as the presence of ≥ 3 of the following 5 components, as recommended by the Adult Treatment Panel III [1]: 1. Low serum HDL cholesterol (<40 mg/dl in men and < 50 mg/dl in women); 2. High serum triglyceride concentrations (≥150 mg/dl); 3. Elevated blood pressure (≥130/85 mmHg); 4. Impaired fasting glucose (fasting plasma glucose concentrations ≥ 110 mg/dl), and 5. Enlarged waist circumference. The cutoff for waist circumference was adopted from the new description of abdominal obesity for Iranian adults, i.e. 95 cm for both genders [21].

### Statistical analysis

The Statistical Package for Social Science (version 15.0; SPSS Inc, Chicago IL) was used for all statistical analyses. Significant differences in characteristics and dietary intakes in the categories of total fat intake and SFA using joint categories of higher (≥ median) versus lower (< median) intakes were evaluated using one-way analysis of variance for continuous variables and values were reported as Mean (SD). Chi-square test was used to detect any significant differences in the distribution of participants across quartile categories with regard to qualitative variables.

Odds Ratio (ORs) and their 95 % confidence intervals was estimated for the MetS and its components, according to both unsaturated fatty acids (MUFA and PUFA) and SFA using joint categories of higher median intake versus lower median intakes [22], by multivariable logistic regression models. In all multivariate models, subjects with lower median intakes of both unsaturated fatty acids (MUFA and PUFA) and SFA were considered as the reference.

## Result

Of 4677 study participants, 44.4 % were male and 55.6 % were female, with a mean age (SD) of 41.7 (13.9) years and had mean BMI of 27.2 (4.7) kg/m^2^. The reported mean (SD) daily intakes were: Total fat 29.9 (6.1) % of total energy intake; SFA 10.2 (2.8) % of total energy intake; MUFA 10.2 (2.8) % of total energy intake and PUFA 6.1 (1.9) % of total energy intake. Baseline characteristics and dietary intakes of the participants according to both total fat and SFA intakes are shown in Table 1. Compared to participants with lower intakes of both SFA and total fat, those with high intake of both SFA and total fat were slightly younger, while there were no significant differences in the physical activity, smoking status, and BMI. Those subjects with high intakes of both SFA and total fat, consumed less carbohydrate, fiber, fruit, vegetable, whole grain, legume and more total fat and SFA, hydrogenated vegetable oils and non hydrogenated vegetable oils (Table 1).

**Table 1:** Characteristics and dietary intakes of participants of the Tehran Lipid and Glucose study, according to Total fat and SFA intakes

	SFA < median		SFA ≥ median		
	Total fat < median	Total fat ≥ median	Total fat < median	Total fat ≥ median	*P* ^*^
Participants (n)	1255	1097	1010	1315	
Age (y)	44.3 ± 14.4^a^	42.3 ± 14.1	40.3 ± 13.4	39.7 ± 13.1	<0.005
Women (%)	46.7	56.2	53.7	65.2	<0.005
Physical activity (%)					
Light	64.1	65.1	64.3	62.3	0.07
Moderate	16.9	17.0	16.3	17.1	
Heavy	19.0	17.9	19.4	20.6	
Current smoker (%)	7.9	8.2	8.1	8.3	0.90
BMI (kg/m2)	27.5 ± 4.6	27.3 ± 4.8	27.1 ± 4.8	27.1 ± 4.8	0.06
Dietary intake^b^					
Total energy intake (Kcal/d)	2329 ± 19.5	2447 ± 21.0	2287 ± 21.7	2425 ± 19.9	0.09
Protein (% of total energy intake)	15.2 ± 0.2	14.3 ± 0.2	16.0 ± 0.2	15.1 ± 0.2	<0.005
Carbohydrate (% of total energy intake)	64.0 ± 0.2	59.2 ± 0.2	57.6 ± 0.2	54.0 ± 0.2	<0.005
Total fat (% of total energy intake)	26.1 ± 0.3	32.8 ± 0.4	31.0 ± 0.4	38.3 ± 0.3	<0.005
SFA (% of total energy intake)	7.5 ± 0.3	7.9 ± 0.4	11.8 ± 0.4	12.4 ± 0.4	<0.005
MUFA (% of total energy intake)	7.7 ± 0.3	10.1 ± 0.4	9.7 ± 0.4	12.7 ± 0.3	<0.005
PUFA(% of total energy intake)	4.4 ± 0.3	7.2 ± 0.4	4.5 ± 0.4	7.9 ± 0.3	<0.005
Cholesterol (mg/d)	186 ± 3.1	185 ± 3.2	273 ± 3.4	258 ± 3.1	<0.005
Fiber (g/d)	30.4 ± 15.2	29.0 ± 15.0	24.1 ± 12.8	20.6 ± 12.5	<0.005
Fruit	252 ± 7.6	279 ± 8.2	285 ± 8.5	226 ± 7.8	<0.005
Vegetables	278 ± 5.1	278 ± 5.7	302 ± 5.5	264 ± 5.2	<0.005
Meat, poultry and fish	60.9 ± 1.2	65.6 ± 1.4	61.5 ± 1.3	63.2 ± 1.3	<0.005
Whole grain	179 ± 3.0	133 ± 3.3	144 ± 3.2	111 ± 3.0	<0.005
Refine grain	351 ± 4.2	291 ± 4.7	239 ± 4.5	279 ± 4.3	<0.005
Dairy products	362 ± 5.8	558 ± 6.5	293 ± 6.3	449 ± 6.0	<0.005
Legumes	46.8 ± 1.2	55.8 ± 1.2	52.8 ± 1.2	42.3 ± 1.2	<0.005
Nuts	5.6 ± 0.3	5.8 ± 0.3	10.6 ± 0.3	9.9 ± 0.3	<0.005
Hydrogenated vegetable oils	31.5 ± 0.3	40.2 ± 0.3	35.1 ± 0.4	49.5 ± 0.5	<0.005
Non-hydrogenated vegetable oils	19.2 ± 0.7	21.5 ± 0.6	18.5 ± 0.5	23.8 ± 0.5	<0.005

Levels of physical activity were expressed as metabolic equivalent hours per week (METs h/week) and categorized as light (<3 METs h/week), moderate (3–6 METs h/week) and heavy (>6 METs h/week)

Grouped according to < or ≥ median of both Total fat (29.5 % of total energy) and SFA (9.5 % of total energy), with the reference group intake being < median intakes of both MUFA and SFA

*BMI* body mass index, *SFA* saturated fatty acid, *MUFA* monounsaturated fatty acid

^*^ *P* value was compared the characteristics across quartiles, using analysis of ANOVA (for age, BMI and dietary intake) and chi-square test (for categorized variables)

^a^Values are Mean (SE), except for variables determined

^b^Dietary intake were adjusted for age, gender and total energy intake using the ANCOVA analysis, except for total energy intake (adjusted for age and gender)

Table 2 showed the odds ratio of the MetS and its components according to intakes of both SFA and total fat intakes. After adjustment for lifestyle and dietary confounders, high SFA intake (≥9.5 % of total energy) was associated with a 39 % higher prevalence of MetS, among subjects with lower total fat intake (95 % CI: 1.11-1.74) and **22** % higher among subjects with a higher total fat intake (95 % CI: 1.05-1.55), compared with subjects with lower intakes of both. Also the combination of a high SFA and total fat intakes was associated with abnormal glucose homeostasis, elevated blood pressure, and high serum triglyceride concentrations after adjustment for confounding factors.

**Table 2:** Odds ratio and 95 % confidence interval for metabolic syndrome and its components according to total fat and SFA intakes among participants of the Tehran Lipid and Glucose study^a^

	SFA < median		SFA ≥ median	
	Total fat < median	Total fat ≥ median	Total fat < median	Total fat ≥ median
Enlarged waist circumference				
Model 1	1	1.35 (1.11-1.65)	1.57 (1.37-1.80)	1.40 (1.16-1.67)
Model 2	1	1.24 (1.00-1.54)	1.40 (1.17-1.69)	1.30 (1.07-1.57)
Model 3	1	0.99 (0.71-1.37)	1.26 (0.96-1.66)	1.37 (1.02-1.83)
Low serum HDL cholesterol				
Model 1	1	1.10 (0.91-1.34)	1.07 (0.93-1.22)	1.00 (0.83-1.19)
Model 2	1	1.01 (0.81-1.25)	0.90 (0.74-1.10)	0.95 (0.78-1.14)
Model 3	1	0.97 (0.78-1.21)	0.86 (0.71-1.04)	0.91 (0.75-1.11)
Abnormal glucose homeostasis				
Model 1	1	0.94 (0.74-1.19)	1.31 (1.13-1.53)	1.50 (1.23-1.84)
Model 2	1	0.97 (0.75-1.25)	1.32 (1.07-1.63)	1.38 (1.11-1.70)
Model 3	1	0.87 (0.67-1.14)	1.22 (0.98-1.52)	1.32 (1.06-1.65)
Elevated blood pressure				
Model 1	1	1.31 (1.04-1.64)	1.29 (1.05-1.60)	1.49 (1.27-1.74)
Model 2	1	1.29 (1.01-1.65)	1.22 (0.98-1.52)	1.44 (1.17-1.77)
Model 3	1	1.17 (0.91-1.52)	1.15 (0.91-1.45)	1.32 (1.06-1.64)
High serum triglyceride concentrations				
Model 1	1	1.24 (1.00-1.53)	1.18 (0.97-1.44)	1.73 (1.50-2.00)
Model 2	1	1.06 (0.84-1.34)	1.10 (0.90-1.35)	1.42 (1.16-1.73)
Model 3	1	0.99 (0.77-1.27)	1.04 (0.84-1.29)	1.36 (1.10-1.68)
Metabolic syndrome				
Model 1	1	1.16 (0.93-1.45)	1.66 (1.44-1.93)	1.40 (1.15-1.71)
Model 2	1	1.09 (0.86-1.38)	1.50 (1.23-1.83)	1.29 (1.10-1.59)
Model 3	1	0.92 (0.70-1.21)	1.39 (1.11-1.74)	1.22 (1.05-1.55)

Model 1 was crude

Model 2 was adjusted for gender, age, smoking status, physical activity, total energy intake, percentage of energy from carbohydrate, percentage of energy from protein, percentage of energy from polyunsaturated fatty acid, total fiber, and cholesterol

Model 3 was further adjusted for BMI

*SFA* saturated fatty acid

^a^Grouped according to < or ≥ median of both total fat (29.5 % of total energy) and SFA (9.5 % of total energy), with the reference group intake being < median intakes of both PUFA and SFA

ORs of the MetS and its components according to intakes of both MUFA and SFA is shown in Table 3. After adjustment for confounding factors, high SFA intake (≥9.5 % of total energy) was associated with higher prevalence of abnormal glucose homeostasis and MetS, whether MUFA intakes was lower or higher than the median intake. Also the combination of a high SFA intakes and a low MUFA intakes was associated with high serum triglyceride concentrations, after adjustment for confounding factors.

**Table 3:** Odds ratio and 95 % confidence interval for metabolic syndrome and its components according to MUFA and SFA intakes among participants of the Tehran Lipid and Glucose study^a^

	SFA < median		SFA ≥ median	
	MUFA < median	MUFA ≥ median	MUFA < median	MUFA ≥ median
Enlarged waist circumference				
Model 1	1	1.08 (0.91-1.28)	1.60 (1.37-1.87)	1.34 (1.14-1.58)
Model 2	1	1.02 (0.85-1.22)	1.38 (1.14-1.68)	1.21 (1.01-1.45)
Model 3	1	0.91 (0.70-1.20)	1.25 (0.94-1.68)	1.24 (0.94-1.63)
Low serum HDL cholesterol				
Model 1	1	0.97 (0.82-1.14)	1.05 (0.90-1.23)	0.97 (0.83-1.14)
Model 2	1	0.94 (0.79-1.12)	0.90 (0.74-1.10)	0.88 (0.74-1.06)
Model 3	1	0.93 (0.78-1.11)	0.87 (0.71-1.05)	0.86 (0.72-1.03)
Abnormal glucose homeostasis				
Model 1	1	0.90 (0.73-1.10)	1.32 (1.10-1.58)	1.33 (1.11-1.60)
Model 2	1	0.96 (0.77-1.18)	1.39 (1.12-1.74)	1.30 (1.06-1.59)
Model 3	1	0.92 (0.74-1.14)	1.30 (1.03-1.63)	1.26 (1.02-1.55)
Elevated blood pressure				
Model 1	1	0.98 (0.80-1.19)	1.43 (1.20-1.71)	1.23 (1.02-1.48)
Model 2	1	0.95 (0.77-1.17)	1.30 (1.05-1.63)	1.13 (0.92-1.39)
Model 3	1	0.91 (0.73-1.13)	1.20 (0.95-1.51)	1.08 (0.87-1.33)
High serum triglyceride concentrations				
Model 1	1	1.12 (0.94-1.35)	1.76 (1.49-2.07)	1.38 (1.16-1.64)
Model 2	1	1.01 (0.83-1.22)	1.36 (1.10-1.68)	1.19 (0.98-1.44)
Model 3	1	1.00 (0.81-1.22)	1.30 (1.05-1.63)	1.16 (0.95-1.42)
Metabolic syndrome				
Model 1	1	0.97 (0.80-1.17)	1.64 (1.38-1.94)	1.39 (1.17-1.67)
Model 2	1	0.93 (0.76-1.14)	1.44 (1.17-1.78)	1.26 (1.14-1.53)
Model 3	1	0.87 (0.69-1.08)	1.33 (1.05-1.68)	1.22 (1.05-1.52)

Model 1 was crude

Model 2 was adjusted for gender, age, smoking status, physical activity, total energy intake, percentage of energy from carbohydrate, percentage of energy from protein, percentage of energy from polyunsaturated fatty acid, total fiber, and cholesterol

Model 3 was further adjusted for BMI

*SFA* saturated fatty acid, *MUFA* monounsaturated fatty acid

^a^Grouped according to < or ≥ median of both MUFA (9.6 % of total energy) and SFA (9.5 % of total energy), with the reference group intake being < median intakes of both PUFA and SFA

Table 4 showed the odds ratio of MetS and its components according to both PUFA and SFA intakes. After adjustment for lifestyle and dietary confounders, higher SFA intake (≥9.5 % of total energy) was associated with higher prevalence of MetS, in both participants with higher and lower median intakes of PUFA. Also high SFA intake (≥9.5 % of total energy) was associated with higher prevalence of abnormal glucose homeostasis, whether PUFA intakes was lower or higher.

**Table 4:** Odds ratio and 95 % confidence interval for metabolic syndrome and its components according to PUFA and SFA intakes among participants of the Tehran Lipid and Glucose study^a^

	SFA < median		SFA ≥ median	
	PUFA < median	PUFA ≥ median	PUFA < median	PUFA ≥ median
Enlarged waist circumference				
Model 1	1	1.08 (0.91-1.28)	1.06 (0.71-1.27)	1.24 (0.94-1.63)
Model 2	1	1.02 (0.85-1.22)	1.04 (0.70-1.25)	1.21 (0.88-1.45)
Model 3	1	0.91 (0.70-1.20)	0.99 (0.69-1.22)	1.04 (0.70-1.58)
Low serum HDL cholesterol				
Model 1	1	0.97 (0.83-1.14)	1.05 (0.90-1.23)	0.97 (0.82-1.14)
Model 2	1	0.88 (0.74-1.06)	0.90 (0.74-1.10)	0.94 (0.79-1.12)
Model 3	1	0.86 (0.72-1.03)	0.87 (0.71-1.05)	0.93 (0.78-1.11)
Abnormal glucose homeostasis				
Model 1	1	0.90 (0.73-1.10)	1.32 (1.15-1.58)	1.37 (1.11-1.60)
Model 2	1	0.96 (0.77-1.18)	1.26 (1.09-1.44)	1.30 (1.06-1.59)
Model 3	1	0.92 (0.74-1.14)	1.15 (1.06-1.33)	1.26 (1.02-1.55)
Elevated blood pressure				
Model 1	1	1.23 (0.98-1.48)	1.43 (1.20-1.71)	0.98 (0.80-1.19)
Model 2	1	1.13 (0.92-1.39)	1.30 (0.95-1.63)	0.95 (0.77-1.17)
Model 3	1	1.08 (0.87-1.33)	1.20 (0.85-1.51)	0.91 (0.73-1.13)
High serum triglyceride concentrations				
Model 1	1	1.38 (1.16-1.64)	1.26 (0.79-2.07)	1.12 (0.94-1.35)
Model 2	1	1.19 (0.98-1.44)	1.16 (0.70-1.68)	1.01 (0.83-1.22)
Model 3	1	1.16 (0.95-1.42)	1.10 (0.55-1.63)	1.00 (0.81-1.22)
Metabolic syndrome				
Model 1	1	0.97 (0.80-1.17)	1.64 (1.38-1.94)	1.39 (1.17-1.67)
Model 2	1	0.93 (0.76-1.14)	1.44 (1.17-1.78)	1.26 (1.10-1.53)
Model 3	1	0.87 (0.69-1.08)	1.33 (1.05-1.68)	1.22 (1.05-1.52)

Model 1 was crude

Model 2 was adjusted for gender, age, smoking status, physical activity, total energy intake, percentage of energy from carbohydrate, percentage of energy from protein, percentage of energy from polyunsaturated fatty acid, total fiber, and cholesterol

Model 3 was further adjusted for BMI

*SFA* saturated fatty acid, *PUFA* polyunsaturated fatty acid

^a^Grouped according to < or ≥ median of both PUFA (5.6 % of total energy) and SFA (9.5 % of total energy), with the reference group intake being < median intakes of both PUFA and SFA

## Discussion

The aim of the current study was to investigate whether the background intake of total dietary fat, MUFA and PUFA modulates the effects of SFA on the MetS and its components. Our findings showed that among Tehranian adults, dietary SFA was associated with high prevalence of the MetS and intake of total dietary fat, MUFA and PUFA (higher or lower than the median) did not affect the this association. Also the combination of a high SFA and total fat intakes was associated with abnormal glucose homeostasis, elevated blood pressure, and high serum triglyceride concentrations after adjustment for confounding factors.

In prospective studies, dietary SFA has been considered a cardiometabolic risk factors [5, 6], but review of the current evidence indicates surprisingly controversial findings; some report no effect [7, 8], while a few found beneficial effects of dietary SFA on chronic disease [9, 10]. Types of dietary fats tend to be intercorrelated and therefore background intakes of dietary MUFA and PUFA may affect the association between dietary SFA and cardiometabolic risk factors [13, 23]. In the only large cohort study with a 14 year follow up, SFA intake was positively associated with chronic heart disease, but this association was attenuated and was no longer significant after adjustment for intakes of polyunsaturated fat [23]. A recent prospective study, which investigated the interaction between nutrients and risk of progression of coronary atherosclerosis, found SFA intake to be associated with less progression of coronary atherosclerosis, only significant among subjects consuming less MUFA [10]; the finding was contrary to ours showing that intake of SFA increased the risk of MetS, independent of total dietary fat and MUFA and PUFA [10, 23].

Results on the replacement of MUFA with SFA on cardiometabolic risk factors were inconsistent [12, 24–26]. The effect of substation of SFA by MUFA on cardiometabolic risk factors may be modulated by total dietary fat [12]. In the KANWN multicenter study, after categorizing the subjects by the median energy fat percentage, in the low fat intake group, high MUFA diet increased insulin sensitivity by 20 %, compared to the high SFA diet; however in the high fat diet, no significant difference was found between the effect of the high SFA and high MUFA diet on insulin sensitivity [12]. An observational study also found that changes in insulin resistance were modified by total fat intake, with an improvement in subjects with total fat intake < 35.5% of total energy and no association in subjects with total fat intake > 35.5% of total energy [27], findings consistent with our results. Our findings was showed dietary SFA to be associated with a higher prevalence of abnormal glucose homeostasis, elevated blood pressure, and high serum triglyceride concentrations, only among subject with high intake of total dietary fat.

Our findings show high prevalence of MetS and abnormal glucose homeostasis among subjects with high intake of SFA, independent of dietary PUFA. Consistent with our results, epidemiologic and experimental studies have shown no significant interaction between SFA and PUFA [10, 11]. In a clinical trial study, fish oil had a beneficial effect on some lipid profiles, regardless of whether the diet containing high or low amounts of SFA [11].

Our study has several strengths. In the current study, we evaluate whether total dietary fat, PUFA and MUFA intakes influence relations between SFA and Mets and its components. We found that the association between the SFA intake and some components of MetS is modulated by total dietary fat, as suggested by some previous studies [12, 27]. Also, our findings suggest that dietary SFA was associated with MetS, independent of PUFA and MUFA intake, which may have important implications for MetS prevention in populations.

Some limitations should be considered, one being the use of UASD FCT to determine the intakes of PUFA, MUFA and SFA, because of not having a complete Iranian FCT. Given the cross-sectional design, we could not determine causality between interaction of different type of fatty acids and the MetS and its components. Future studies using longitudinal data are needed to determine these effects. In addition, this study included only healthy adults, and our findings cannot be extrapolated to other populations.

## Conclusion

Our findings indicate that SFA intakes were positively associated with the prevalence of MetS, independent of total dietary fat, MUFA and PUFA intake. However total dietary fat may modulate the association between SFA and some components of MetS, results emphasizing that both the quality and quantity of dietary fat are relevant with Mets and cardiometabolic risk factors.
